# Large fungating basal cell carcinoma of the dorsum of the foot: A case report

**DOI:** 10.1002/ccr3.1730

**Published:** 2018-09-05

**Authors:** Rishi Mamtani, Kamal Addagatla, Elgida R. Volpicelli, Xiang Da (Eric) Dong, Matthew Juriga

**Affiliations:** ^1^ Department of Surgery Stamford Hospital Affiliate of the Columbia University College of Physicians and Surgeons Stamford Connecticut; ^2^ Department of Pathology Stamford Hospital Affiliate of the Columbia University College of Physicians and Surgeons Stamford Connecticut; ^3^ Department of Podiatry Stamford Hospital Affiliate of the Columbia University College of Physicians and Surgeons Stamford Connecticut

**Keywords:** basal cell carcinoma, cryotherapy, full‐thickness skin graft, recurrence, wide excision

## Abstract

Basal cell carcinoma is the most common skin cancer, but may present as anatomically and pathologically unique variants. A careful understanding of the pathophysiology, meticulous preoperative planning, and the use of unique reconstructive techniques to preserve function and cosmesis are key in achieving a satisfactory oncologic result.

## 1 INTRODUCTION

We report a rare presentation of a large, fungating basal cell carcinoma on the dorsum of the foot. Management involved surgical excision with careful planning, with integration of reconstructive techniques to preserve function and cosmesis while accomplishing the required oncologic resection.

Basal cell carcinoma (BCC) is the most common form of skin cancer, classically presenting on areas of maximal sun exposure, such as the face. Presentation of this type of cancer in a location such as the dorsum of the foot is exceedingly rare. It is important to be cognizant of this unique presentation, and the associated steps of treatment to preserve function and cosmesis, while optimizing oncologic outcome.

## 2 CASE REPORT

**Figure 1:**
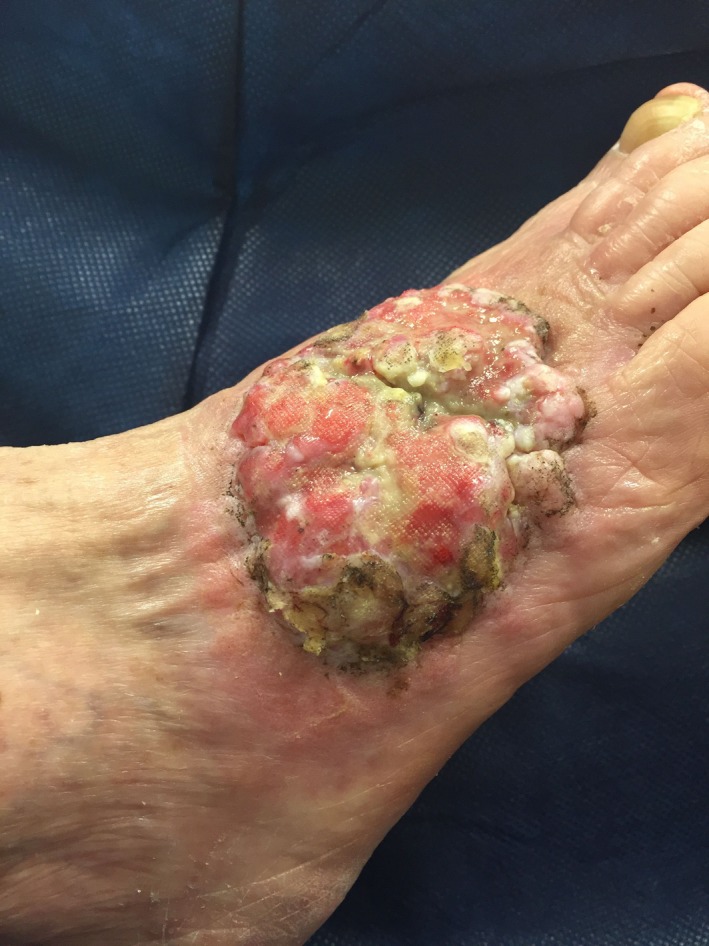
Preoperative image of large fungating basal cell carcinoma on the dorsum of the foot, measuring 8 × 8 × 0.6 cm

**Figure 2:**
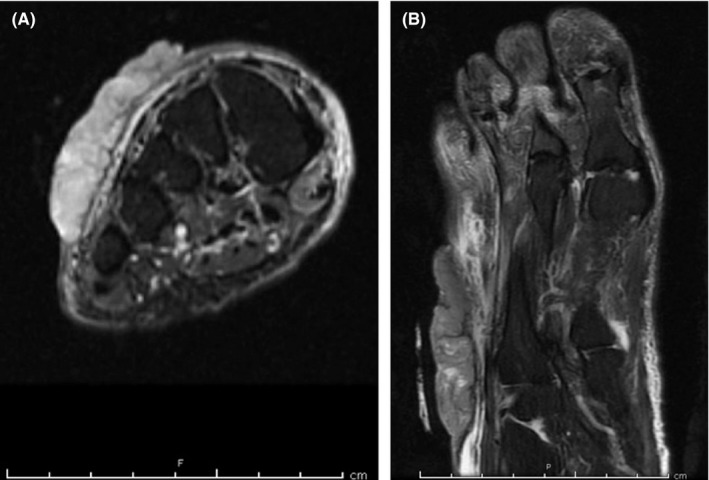
Preoperative magnetic resonance imaging of the lesion in (A) T2‐weighted and (B) short‐tau inversion recovery sequencing

An 85‐year‐old female with a past medical history of diabetes mellitus, hypertension, and hyperlipidemia underwent surgical consultation in October 2015 for an incidentally found right foot mass subsequent to an elective gynecologic procedure. The patient first noticed a small lesion on the dorsum of her right foot approximately 12 years ago. She never sought medical attention for this slowly growing and asymptomatic lesion. However, this lesion had been growing more rapidly and had become foul smelling over the past 3 weeks. She denied any pain, paresthesias, weight loss, or recent trauma to the foot. She also denied any history of smoking or significant sun exposure. Her only surgical history included a recent total abdominal hysterectomy and left salpingo‐ophorectomy. On physical examination, she was a well‐nourished Caucasian female who was found to have a large exophytic, fungating 8.0 × 8.0 × 0.6 cm malodorous mass on the dorsum of her right foot (Figure 1). This mass was insensate to light touch with no surrounding erythema. She had palpable dorsalis pedis and posterior tibial pulses with full range of motion of that foot. The left lower extremity was benign in examination, with no masses or lesions. A thorough dermatologic examination did not reveal any other concerning lesions. A punch biopsy of this mass was consistent with BCC. Subsequent magnetic resonance imaging (MRI) showed a large soft tissue irregularity surrounding the second to fifth extensor tendons (Figure 2). Given these findings, the patient underwent a wide local excision of this BCC with full‐thickness skin graft for coverage.

**Figure 3:**
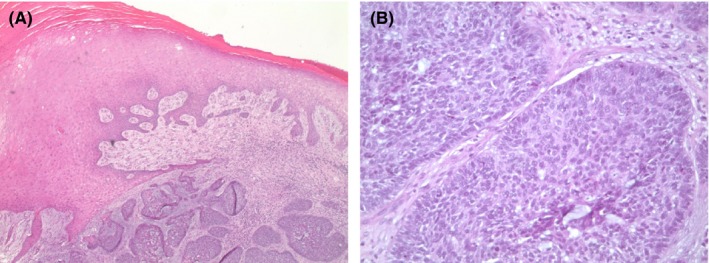
Histology images on low‐ and high‐power magnification, prepared by hematoxylin and eosin staining. Low‐power image (A) reveals reactive epidermal acanthosis with underlying nodular blue islands composed of hyperchromatic keratinocytes embedded in a fibromyxoid stroma. High‐power image (B) reveals retraction artifact between nodular blue islands and surrounding stroma, hyperchromatic nuclei, high nuclear/cytoplasmic ratio, and peripheral palisading

**Figure 4:**
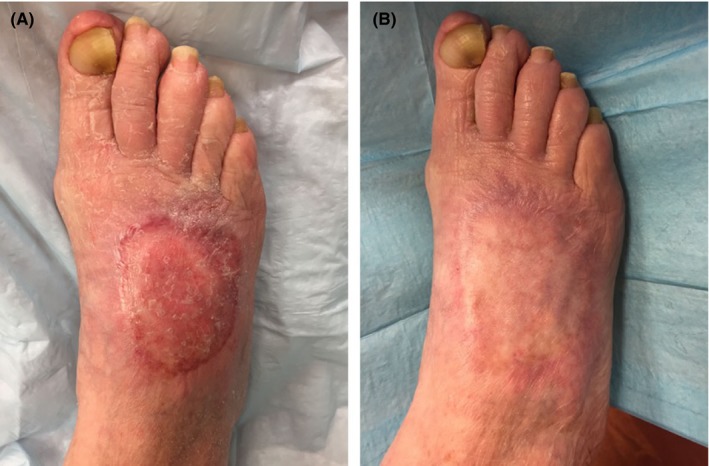
Postoperative wound evaluations at (A) seven and (B) 12 mo

The lesion was excised in its entirety with a 5‐mm circumferential margin beyond the extent of visible tumor. This incision was carried down directly to the fascia overlying the extensor digitorum longus tendons. Intraoperative pathologic evaluation noted a negative deep margin; therefore, resection of extensor tendons was not required. The specimen was sent to pathology en bloc revealing an 8.2 × 6.4 × 1.4 cm lesion consistent with BCC, nodular subtype with clear margins (Figure 3). The dorsalis pedis artery was clearly identified and safely avoided during the procedure. A full‐thickness skin graft (FTSG) measuring 7 × 10 cm was obtained from the right lower abdomen. Multiple small incisions were made approximately 1 cm apart on the FTSG to allow for egress of fluid. The FTSG was then tacked onto the wound bed using 5‐0 chromic sutures. The wound was bandaged with nonadherent Xeroform dressing, bulky gauze fluffs, a compression wrap, and placed in a posterior splint for immobilization. The patient was maintained on bed rest with extremity elevation until postoperative day 3 at which point she was allowed to ambulate with toe‐touch weight bearing precautions on her right lower extremity. Dressings were removed on postoperative day 4, revealing a healthy and viable graft. She had palpable pedal pulses and intact motor and sensory function. She was discharged on postoperative day 4 with instructions to continue toe‐touch weight bearing precaution on her right foot with continued outpatient follow‐up. At 1‐year follow‐up, her wound had fully recovered without complication (Figure 4), and at 2‐year surveillance, she remains without any recurrence of disease.

## 3 DISCUSSION

Basal cell carcinoma is the most common malignancy in the world with a lifetime risk of developing disease estimated to be 1 in 5.^1^ BCC occurs more frequently in men, and its incidence increases with age.^1^ However, these tend to occur most frequently on the head and neck and are much less common on the trunk and extremities. A presentation on the dorsum of the foot is an exceedingly rare occurrence.

The pathogenesis of BCC has been identified to originate from an increase in gene expression of the Sonic hedgehog signaling pathway.^1^ Sun exposure is known as the most important environmental risk factor for developing BCC.^1^, ^2^ A positive correlation has been observed between BCC rates and geographic proximity to the equator, which can be explained by higher ultraviolet (UV) exposure at lower latitudes.^1^, ^2^ Other risk factors include prior radiotherapy,^3^ arsenic exposure,^4^ and immunosuppression.^1^ The unusual location on the foot, an area often covered, combined with the absence of any known predisposing risk factors with the exception of well‐controlled diabetes, made this an anomalous diagnosis in our patient. Along with BCC, the differential diagnosis of such a skin lesion includes, but is not limited to, inflammatory or infectious processes, Bowen's disease, squamous cell carcinoma, Merkel cell carcinoma, and amelanotic melanoma.^1^

Pathologic examination is required for definitive diagnosis of BCC. Histopathology shows intensely basophilic aggregations of basaloid keratinocytes with palisading nuclei. Further histologic characterization can help to distinguish tumors as either indolent (ie, nodular or superficial) or aggressive (ie, morpheaform, infiltrative, micronodular, or basosquamous).^1^ Nodular BCC is the most common subtype accounting for up to 80% of cases that characteristically present as a pearly, raised lesion on the head.^5^ Superficial BCC comprises of up to 15% of cases and, in contrast, presents as a pink, macular lesion showing predilection for the trunk and extremities. Lastly, the morpheaform subtype, which appears plaque‐like, is the most rare and locally destructive of the three subtypes.^5^

The mainstay of treatment of BCC involves complete excision of the tumor. Risk of recurrence after excision is higher with size >2 cm, poorly defined borders, aggressive‐type pathologies, and in cases of immunosuppressed patients or those with a history of radiotherapy.^1^ Lesions at low risk for recurrence may be managed with either electrodessication and curettage, or surgical excision, as was done for our patient. Patients at higher risk for recurrence may particularly benefit from the staged technique of Mohs micrographic surgery, which utilizes frozen section evaluation of surgical margins. This has been shown to have the lowest rate of recurrence of 1.4% at 5 years^6^ and is preferable in locations requiring tissue conservation such as the face.^1^ As 5‐year cure rates have been shown to exceed 95% with 4‐ to 5‐mm surgical margins for BCC on the trunk, extremities, or head and neck, these are the most commonly recommended margins for resection of uncomplicated BCC.^7^, ^8^, ^9^ Alternative treatment options include the new oral inhibitors of the sonic hedgehog pathway (vismodegib and sonidegib) reserved for metastatic or locally aggressive disease, topical imiquimod cream, photodynamic therapy, cryosurgery, and radiotherapy; however, these treatment modalities are less efficacious.^1^, ^10^ A large review of 40 studies recommends surgical excision as the standard of care to ensure the lowest recurrence rates and the best cosmetic outcomes.^6^ The reported 5‐year recurrence rate for primary BCC after oncologic resection is 10%, with most recurrences seen within 4‐12 months after initial treatment.^10^

There are limited case reports in the literature reporting similar BCC presentation in the foot, although our lesion appears to be the largest of this group.^11^, ^12^, ^13^ One of the reported cases appears to have recurred as metastatic disease 2.5 years later, with metastasis to inguinal and pelvic lymph nodes originating from a primary lesion on the dorsum of the foot.^11^, ^12^, ^13^ In this case, the large area of involvement and invasion down to fascia required postexcisional flap coverage with a full‐thickness skin graft.^11^, ^12^, ^13^ Recurrence of BCC within a skin graft is rare, but has been reported,^14^ and continued surveillance for recurrence remains essential even in such cases requiring complex reconstructions.

In summary, our unique case emphasizes the importance of including BCC in the differential diagnosis of a large lesion even in the unusual location such as the dorsum of the foot. Although sun exposure is noted to be the leading risk factor for the development of BCC, clearly genetic mutations and predispositions can result in this disease. Surgical excision of BCC remains the standard of care for treatment of BCC and may at times require reconstructive surgical procedures to preserve function after extensive oncologic resection.

## CONFLICT OF INTEREST

None declared.

## AUTHORSHIP

RM, and KA: collected the information, reviewed the case, and wrote the manuscript. ERV, XDD, and MJ: reviewed the manuscript. ERV: involved in pathology review. XDD: involved in primary surgical attending. MJ: involved in primary podiatric attending.
